# Pain-Specific Resilience in People Living With HIV and Chronic Pain: Beneficial Associations With Coping Strategies and Catastrophizing

**DOI:** 10.3389/fpsyg.2019.02046

**Published:** 2019-09-06

**Authors:** Cesar E. Gonzalez, Jennifer I. Okunbor, Romy Parker, Michael A. Owens, Dyan M. White, Jessica S. Merlin, Burel R. Goodin

**Affiliations:** ^1^Department of Psychology, University of Alabama at Birmingham, Birmingham, AL, United States; ^2^Department of Anesthesia and Perioperative Medicine, University of Cape Town, Cape Town, South Africa; ^3^Department of Medicine, Divisions of General Internal Medicine and Infectious Diseases, University of Pittsburgh, Pittsburgh, PA, United States

**Keywords:** HIV-human immunodeficiency virus, chronic pain, resilience (psychological), coping, catastrophizing

## Abstract

**Objective:**

Chronic pain is increasingly recognized as a common and disabling problem for people living with HIV (PLWH). In a recent systematic review of psychosocial factors associated with chronic pain in PLWH, it was reported that very few studies to date have examined protective psychological factors that might help mitigate chronic pain for PLWH. The current study examined pain-specific resilience in relation to clinical and experimental pain, as well as pain coping in PLWH and chronic pain. Pain-specific resilience specifically refers to the ability to maintain relatively stable, healthy levels of psychological and physical functioning in the face of ongoing and persistent pain.

**Methods:**

A total of 85 PLWH (mean CD4 = 643; 13% detectable viral load ≥200; 99% on antiretroviral therapy) who met criteria for chronic pain (>3 consecutive month’s duration) were enrolled. Medical records were reviewed to confirm clinical data. All participants provided sociodemographic information prior to completing the following validated measures: Pain Resilience Scale (PRS), Coping Strategies Questionnaire-Revised (CSQ-R), Center for Epidemiologic Studies – Depression Scale (CES-D), and the Brief Pain Inventory – Short Form (BPI-SF). They then completed a quantitative sensory testing battery designed to assess tolerance for painful heat and cold stimuli.

**Results:**

In adjusted multiple regression models controlling for covariates, greater pain-specific resilience was significantly associated with less pain interference (*p* = 0.022) on the BPI-SF, less pain catastrophizing (*p* = 0.002), greater use of distraction (*p* = 0.027) and coping self-statements (*p* = 0.039) on the CSQ-R, as well as significantly greater heat pain tolerance (*p* = 0.009). Finally, results of a parallel multiple mediation model demonstrated that the effect of pain-specific resilience on heat pain tolerance was indirectly transmitted through less pain catastrophizing (95% confidence interval:0.0042 to 0.0354), but not use of distraction (95% confidence interval: −0.0140 to 0.0137) or coping self-statements (95% confidence interval: −0.0075 to 0.0255).

**Conclusion:**

The findings suggest that pain-specific resilience may promote adaptation and positive coping in PLWH and chronic pain.

## Introduction

Due to advancements in antiretroviral therapy (ART), people living with HIV (PLWH) who are connected to care and adhere to their medication regimens can achieve near normal life expectancies (Negin et al., 2012; O’Keefe et al., 2013). However, living longer with HIV is often accompanied by an increased likelihood of developing HIV-associated chronic health conditions (Pitts et al., 2005). One particularly important health condition that affects aging PLWH is chronic pain (Merlin et al., 2012). Estimates suggest that chronic pain may affect over half of all PLWH throughout their lifetimes (Parker et al., 2014). The experience of chronic pain in PLWH often comes at a high cost, such that it significantly and negatively impacts quality of life (Merlin et al., 2013, 2014). Furthermore, treatment of pain in this population can be difficult due to complicating factors including substance use and psychiatric illness (Tsao and Soto, 2009). Pharmacologic pain treatment options, including opioid medications, have limited efficacy for managing chronic pain for many PLWH (Bruce et al., 2017). Psychological approaches for chronic pain in PLWH have demonstrated initial promise (Merlin et al., 2018); however, the full potential of this treatment modality to yield positive outcomes remains underappreciated given the lack of sufficient research to date focused on psychological contributors to chronic pain in PLWH (Scott et al., 2018).

The extant literature addressing psychological contributors to chronic pain in PLWH has primarily focused on vulnerabilities and risk factors for poor outcomes. Scott et al. (2018) recently published a comprehensive review on this topic indicating that depression, psychological distress, post-traumatic stress and substance use were the psychological factors most associated with negative pain outcomes in PLWH. Only a small number of studies thus far have examined the role of protective psychological factors in relation to chronic pain for PLWH. For example, PLWH and chronic pain may possess lower optimism (Simmonds et al., 2005) and self-efficacy for disease management (Parker et al., 2017) and treatment adherence (Berg et al., 2009) relative to PLWH without chronic pain. In a study by Wadley et al. (2016), PLWH and chronic pain reported significantly lower levels of resilience compared to PLWH without chronic pain. However, in this same study resilience was not significantly associated with pain severity or interference for PLWH and chronic pain. Despite these equivocal findings, additional research investigating the association of resilience with chronic pain in PLWH appears warranted for two reasons. First, it is well documented that PLWH are often able to remain resilient despite the many hardships they often face (Dale et al., 2014; Emlet et al., 2017). Second, in non-HIV populations with chronic pain, high resilience has been associated with positive responses to pain, adaptive coping styles, and favorable health care and medication utilization patterns (Karoly and Ruehlman, 2006; Sturgeon and Zautra, 2010).

Resilience is broadly conceptualized as the ability to cope with a crisis or adversity while maintaining positive emotional and physical functioning (Joyce et al., 2018). Numerous measures have been developed to assess resilience as a general psychological construct, and indeed these measures have proven effective for predicting adaptation to chronic pain (Ong et al., 2010; Ramírez-Maestre et al., 2012). More recently, it has been suggested that a pain-specific measure of resilience is likely to be better suited for studies examining clinical and experimental pain experiences than a general measure of psychological resilience (Slepian et al., 2016). Pain-specific resilience specifically refers to the ability to maintain relatively stable, healthy levels of psychological and physical functioning in the face of ongoing and persistent pain (Ankawi et al., 2017). In the laboratory setting, high pain-specific resilience has been shown to be associated with less sensitivity to experimental pain stimuli during quantitative sensory testing (QST) (Slepian et al., 2016). Further, a series of clinical studies conducted with chronic pain samples found that high pain-specific resilience was associated with better quality of life and lower pain intensity (Ankawi et al., 2017), as well as greater pain self-efficacy and acceptance (Slepian et al., 2018). It remains to be determined whether a measure of pain-specific resilience might also be associated with clinical and experimental pain in a sample of PLWH and chronic pain.

In studies conducted with non-HIV populations, highly resilient individuals with chronic pain have been shown to report greater positive emotions and less pain catastrophizing compared to their less resilient counterparts (Ong et al., 2010; Sturgeon and Zautra, 2013). Similarly, resilient individuals have been found to engage in more adaptive pain coping strategies, which promote efforts to control pain and to function at a high level in spite of pain (Sturgeon and Zautra, 2010). Adaptive pain coping strategies such as distraction and positive coping self-statements facilitate adaptation to chronic pain while also decreasing sensitivity to experimental pain stimuli (Roditi et al., 2009; Malloy and Milling, 2010; Verhoeven et al., 2011). Based upon the existing literature, it stands to reason that pain-specific resilience may be associated with less pain catastrophizing and greater engagement in adaptive pain coping strategies for PLWH and chronic pain, which in turn would be associated with decreased severity of clinical and experimental pain experiences.

The primary objective of the current study was to examine whether pain-specific resilience was associated with reports of clinical and experimental pain, as well as pain catastrophizing and coping strategies, in PLWH and chronic pain. Three distinct hypotheses were tested. (1) High pain-specific resilience would be significantly associated with lower clinical pain severity and interference, as well as greater tolerance for painful cold and heat stimuli during QST. (2) High pain-specific resilience would be significantly associated with less pain catastrophizing and greater use of pain coping strategies (e.g., distraction). (3) Pain catastrophizing and active pain coping strategies would significantly mediate the effect of pain-specific resilience on clinical and experimental pain.

## Materials and Methods

### Study Design Overview

People living with HIV with chronic pain were recruited via posted flyers from a large, urban HIV clinic in Alabama, United States. that provides comprehensive medical, social, and behavioral services to approximately 3,500 adults (≥18 years) living with HIV. Those interested in study participation were assessed for eligibility during an initial telephone screening. Medical records were then reviewed for each prospective participant to assist with eligibility determination. Eligible participants subsequently presented to the laboratory to complete a single study session. At the beginning of the study session resting blood pressure and core body temperature were recorded for each participant. Blood was then taken from each participant for determination of CD4+ count and viral load. Participants completed a QST battery designed to assess tolerance for thermal pain (heat and cold). Following QST, participants completed standardized self-report questionnaires that assessed pain severity and interference, pain-specific resilience, pain coping, and pain catastrophizing. Sociodemographic information was collected from all participants, and this information included age, natal sex, ethnicity/race, educational attainment, and poverty status. Poverty status was determined through adjusting the recorded annual household income by number of occupants through guidelines put forth by the 2017 United States Department of Health and Human Services (U. S. Federal Poverty Guidelines, 2017).

### Medical Record Review

Medical record reviews were completed to ascertain rates of psychiatric diagnoses among participants, as well as determine whether participants were actively being prescribed antiretroviral therapy (ART). Medical record review also assisted with determining duration of chronic pain and whether participants were actively being prescribed analgesic medications that could affect reported pain and/or responses to QST, particularly opioids (Niesters et al., 2013). Lastly, medical record review was used to confirm participants’ self-reported health history provided during telephone screening. Those PLWH and chronic pain whose medical records corroborated their self-reported health history, and who met study inclusion criteria, were deemed eligible for ongoing participation.

### Participants

A total of 91 PLWH and chronic pain were enrolled into this cross-sectional study. Six participants were disqualified from further participation due to the presence of uncontrolled hypertension, which was a contraindication for the completion of QST. This resulted in a final study sample size of 85 PLWH and chronic pain. Study procedures were approved by the local Institutional Review Board and carried out in accordance with guidelines for the ethical conduct of research. Written informed consent was obtained from each participant prior to the study, and the participants were compensated for their participation.

People living with HIV with chronic pain were included in this study if they reported chronic pain that had persisted for at least three consecutive months and was present on at least half the days in the past 6 months (Treede et al., 2015). Additional inclusion criteria were: age ≥18 years; no evidence of uncontrolled hypertension (i.e., resting blood pressure >150/95); no circulatory disorders (e.g., Raynaud’s disease); no history of cardiac events, no history of stroke, seizures, or other neurological disorders, no history of metabolic disease, no history of cancer and related treatment, and not currently pregnant. Furthermore, participants were excluded from study participation if they demonstrated signs of acute infection (i.e., core body temperature >37.8°C), reported any pain-alleviating surgery within the past year, or receipt of any pain intervention treatment within the past month (e.g., steroid injection).

### Measures

#### Quantitative Sensory Testing

Previous studies have found that the relationship between resilience and responses to QST emerges during prolonged exposures to painful stimuli (Pulvers and Hood, 2013). For this reason, the QST battery in this study was designed to specifically assess tolerance for painful thermal stimuli (heat and cold). Heat pain tolerance (HPTo) refers to the maximum heat stimulus intensity (i.e., temperature, °C) a person is willing to tolerate before discontinuing due to pain. Similarly, cold pain tolerance (CPTo) refers to the maximum duration (i.e., time in seconds) a person is willing to tolerate a cold stimulus prior to discontinuing due to pain. Participants prescribed analgesics including opioids were not asked to abstain from these medications prior to the completion of QST given that temporary withdrawal could alter pain responses (Mao, 2006).

#### Heat Pain Tolerance

HPTo was recorded as the temperature in Celsius at which the participant discontinued the heat stimulus. HPTo was assessed on participants’ ventral forearm using a Medoc Thermal Sensory Analyzer-II (TSA) (Medoc Ltd., Ramat Yishai, Israel) with a 30 × 30-mm-diameter thermode in accordance with an ascending method of limits. From a baseline of 32°C, probe temperature increased at a rate of 0.5°C/s until participants responded by pressing a button on the patient response unit to indicate when they were no longer able to tolerate the pain. Three trials of HPTo were completed separately, and the position of the thermode was altered slightly between trials so that the site of stimulation did not overlap (though it remained on the ventral forearm). The average HPTo across all three trials was computed for use in statistical analysis.

#### Cold Pain Tolerance

For the assessment of cold pain tolerance (CPTo), participants were asked to fully immerse their non-dominant hand up to the wrist in a cold pressor for a maximum of 300 s. The water temperature was maintained at 10°C (±0.050C) by an ARTIC A25 refrigerated bath with an SC150 immersion circulator (Thermo Fisher Scientific, United States) that constantly circulated the water to prevent local warming around the submerged hand. The water temperature was selected based upon our previous work with other PLWH cohorts demonstrating that 10°C was deemed moderately painful and resulted in the most normally distributed range of CPTo. Participants were asked to give pain intensity ratings on a 0 (no pain) to 100 (most intense pain possible) numeric rating scale at 30 and 60 s intervals. Participants were told that they could remove their hand from the cold pressor at any time if the pain became intolerable. The procedure lasted either the full 300 s or until the participant discontinued. Time of hand removal was recorded in seconds and included as an index of CPTo for statistical analysis.

#### Pain Severity and Interference

The Brief Pain Inventory – Short Form (BPI-SF) is a multidimensional pain scale used to assess the severity of pain and its impact on daily functioning (Tan et al., 2004). The questionnaire is composed of four items asking about pain intensity (worst pain, least pain, average pain, and pain right now) over the past 24 h. There are also seven items that assess the degree to which pain interferes with functioning in the following domains: general activity, mood, walking ability, normal work, relations with other people, sleep, and enjoyment of life. The BPI-SF yields two overall scores: a pain severity score and a pain interference score. The pain severity score is the average of the four items asking about worst, least, average, and current pain. Each item is scored from 0 (no pain) to 10 (worst imaginable pain). The pain interference score is the average of the nine items addressing functional impairment. Each item is scored from 0 (does not interfere) to 10 (completely interferes). Higher scores suggest great pain severity and interference. Overall, the BPI-SF in this study had excellent internal consistency (Cronbach’s α = 0.95).

#### Pain-Specific Resilience

The Pain Resilience Scale (PRS) is a 14-item assessment of resilience in the presence of intense or prolonged pain. The assessment has 2 subscales to measure specific domains of resilience: behavioral perseverance and cognitive/affective positivity (Slepian et al., 2016; Ankawi et al., 2017). The behavioral perseverance subscale examines an individual’s ability to continue engaging in behaviors or activity when experiencing pain. The cognitive/affective positivity subscale examines an individual’s ability to maintain positive thoughts and manage negative thoughts or emotions while in pain. Each item is scored from 0 (not at all) to 4 (all the time) to determine the degree to which individuals engage in resiliency resources. The total PRS score results from the summation of response to all 14 items. Higher scores are suggestive of greater pain related resilience. The PRS used in this study possessed excellent internal consistency (Cronbach’s α = 0.94).

#### Pain Coping and Catastrophizing

The Coping Strategies Questionnaire-Revised (CSQ-R) is a 27-item assessment that was utilized to assess participants’ use of cognitive strategies to cope with pain, as well as pain catastrophizing (Rosenstiel and Keefe, 1983). The CSQ-R includes the following subscales representing six cognitive domains: distraction (five-items), ignoring pain sensations (five-items), distancing oneself from pain (four-items), coping self-statements (four-items), praying/hoping (three-items), and catastrophizing (six-items). Each item is scored from 0 (never do that) to 6 (always do that) to indicate how frequently the strategy is engaged in response to pain. Each subscale is scored separately, and higher scores indicate greater engagement in that respective cognitive domain. The CSQ-R in this study had adequate internal consistency (Cronbach’s α = 0.73).

#### Depression

Depressive symptoms were assessed using the Center for Epidemiological Studies Depression Scale (CES-D). This 20-item measure assesses the frequency of experiencing depressive symptoms over the past week (0 – never or rarely, to 3 – most of the time/all the time). Symptoms of depression measured by the CES-D include negative mood, guilt/worthlessness, helplessness/hopelessness, psychomotor retardation, loss of appetite, and sleep disturbance (Radloff, 1977). This measure has been shown to be reliable and valid in general populations, as well as HIV and chronic pain populations (Geisser et al., 1997; Natamba et al., 2014). Responses are summed (0–60), with higher scores indicating greater severity of depression. The CES-D measure used in the current study had good internal consistency (Cronbach’s α=0.88).

#### CD4 and Viral Load

Blood was collected from each participant at the beginning of the study session and sent to the local diagnostics laboratory for quantification of CD4 helper T-cell count and viral load. Absolute CD4 helper T-cell count was quantified as cells/microliter of blood, while viral load was quantified as viruses/microliter. Participants with 200 viruses/microliter of blood or greater were considered to be “detectable.” CD4 and viral load reflect immune health and response to ART therapy, respectively. Each was included in this study to assess whether these aspects of HIV infection were associated with clinical and/or experimental pain.

### Data Organization and Analysis

All data were analyzed using SPSS, version 25 (IBM; Chicago, IL, United States). All participants provided complete demographic (e.g., sex, age) and QST data; however, a small portion of missing data existed for one or more key study variables such as pain-specific resilience and pain coping (≤5% of the total data comprising each measure). Data appeared to be missing at random. A simple data imputation method was completed using the macro for Hot Deck imputation (Myers, 2011). This data imputation method is well validated and accepted in the statistical community and resulted in complete study data for each of the 85 study participants.

Descriptive data for the sample are presented as percentages or as means and standard deviations. Differences across categorical variables were assessed using chi-square tests, while differences on continuous variables were assessed using analysis of variance (ANOVA). Zero order relationships among all study variables were assessed using Pearson correlations. To assess the unique relationships of pain-specific resilience with pain severity and interference, HPTo and CPTo, as well as pain coping and catastrophizing, a series of linear multiple regressions was completed controlling for selected covariates. The PROCESS macro (model 4) created and described by Hayes (2013) for obtaining 95% bootstrapped confidence interval with 5,000 resamples was utilized to test whether catastrophizing and/or any of the pain coping strategies significantly mediated the associations between pain-specific resilience and pain, including pain severity, pain interference, HPTo, and CPTo.

## Results

### Participant Characteristics

Descriptive characteristics for the 85 study participants are presented in Table 1. The mean age of the sample was 49 years. The study population was comprised of 67% men and 33% women. The majority of the study sample was non-Hispanic Black (74%), and lived below the poverty line (85%). The mean CD4 count was 643 cells/mm^3^, 13% had a detectable viral load, and 99% were actively prescribed antiretroviral therapy. Seventeen percent of the study sample were prescribed opioid medications for pain. The most frequently reported locations of chronic pain were low back/hips (46%), legs/feet (25%), widespread (2 + sites) (20%), arms/hands (6%), head (2%), and neck/shoulders (1%). Medical records indicated that 24% of the sample had a pain duration of >3 months but <1 year, 25% >1 year but <5 years, 23% >5 years but <10 years, and 28% >10 years. Average pain severity over the past 24 h was 5.8, while pain interference was 4.5 on the 0–10 numeric rating scale of the BPI-SF.

**TABLE 1:** Participant characteristics (*N* = 85).

**Variable**	**Mean (SD) or Count (%)**	**Range**
**Demographic characteristics**	49 (8.3)	
Age–Years		26–67
Sex		
Males	57(67%)	
Females	28(33%)	
Race		
non-Hispanic Black	63(74%)	
non-Hispanic White	16(19%)	
American Indian	1(1%)	
Multiracial	5(6%)	
Poverty		
Below Poverty Line	72(85%)	
Above Poverty Line	13(15%)	
**Clinical characteristics**		
CD4	643 (324)	62–2,491
Viral load (≥200 copies/mL)		
Undetectable	74(87%)	
Detectable	11(13%)	
Anti-Retroviral Therapy (ART)		
Actively Prescribed	84(99%)	
Not Prescribed	1(1%)	
Opioids		
Actively Prescribed	14(17%)	
Not Prescribed	71(83%)	
**Depressive symptoms**		
CES-D – Depressive Symptoms	21.2 (11.5)	0–53
**Resilience**		
PRS	36.35 (13.55)	0–56
**Coping**		
CSQ-R – Catastrophizing	2.5 (1.4)	0–6
CSQ-R – Distraction	2.6 (1.6)	0–6
**Pain duration**		
>3 months but <1 year	20(24%)	
>1 year but <5 years	22(25%)	
>5 years but <10 years	19(23%)	
>10 years	24(28%)	
**Clinical pain severity and interference**		
BPI-SF – Pain Severity	5.8 (2.4)	0–9.8
BPI-SF – Pain Interference	4.5 (2.8)	0–10
**Experimental pain**		
HPTo (°C)	48.1 (2.2)	38.6–50.5
CPTo (seconds)	163.9 (111.7)	12–300

*CES-D, Center for Epidemiologic Studies-Depression Scale; PRS, Pain-Specific Resilience Scale; CSQ-R, Coping Strategies Questionnaire-Revised; BPI-SF, Brief Pain Inventory-Short Form; HPTo, heat pain tolerance; CPTo, cold pain tolerance.*

### Bivariate Associations and Selection of Covariates

Zero-order Pearson correlations among continuously measured variables are presented in Table 2. Greater pain-specific resilience was significantly correlated with less depressive symptoms (*p* = 0.011) and less pain catastrophizing (*p* < 0.001), greater use of distraction (*p* = 0.007) and coping self-statements (*p* = 0.013), less clinical pain severity (*p* = 0.042) and pain interference (*p* < 0.001), as well as greater HPTo (*p* = 0.014) and CPTo (*p* = 0.041). Greater depressive symptom severity was significantly correlated with increased pain interference (*p* < 0.001) and more pain catastrophizing (*p* < 0.001). Greater pain catastrophizing was significantly correlated with greater clinical pain severity (*p* = 0.034) and pain interference (*p* = 0.001), as well as diminished HPTo (*p* = 0.003) and CPTo (*p* = 0.031). Longer duration of pain was significantly correlated with greater clinical pain severity (*p* < 0.001) and pain interference (*p* = 0.034), as well as less pain-specific resilience (*p* = 0.038). Results from a series of one-way ANOVAs revealed that participants actively prescribed opioids tended to report greater pain interference (*p* = 0.069) than those not receiving opioid medication. A significant sex difference was observed for HPTo (*p* = 0.013), such that males demonstrated diminished HPTo compared to females Age, race, poverty status, and HIV clinical characteristics including CD4+ and detectable viral load, were not significantly associated with any of the key variables of interest.

**TABLE 2:** Zero-order pearson correlations.

**Variable**	**1**	**2**	**3**	**4**	**5**	**6**	**7**	**8**	**9**	**10**	**11**	**12**
(1) PRS	–											
(2) CES-D	–0.279^∗∗^	–										
(3) CSQ-R Catastrophizing	–0.453^∗∗^	0.471^∗∗^	–									
(4) CSQ-R Distancing	0.098	0.030	0.362^∗∗^	–								
(5) CSQ-R Distraction	0.293^∗∗^	−0.264^∗^	0.036	0.486^∗∗^	–							
(6) CSQ-R Ignoring	0.021	–0.037	0.313^∗∗^	0.715^∗∗^	0.525^∗∗^	–						
(7) CSQ-R self-statements	0.267^∗^	–0.285^∗∗^	–0.094	0.215^∗^	0.638^∗∗^	0.404^∗∗^	–					
(8) CSQ-R Praying/Hoping	–0.067	–0.038	0.141	0.162	0.282^∗∗^	0.247^∗^	0.304^∗∗^	–				
(9) BPI-SF pain severity	−0.221^∗^	0.076	0.230^∗^	0.134	–0.072	0.082	–0.138	0.073	–			
(10) BPI-SF Pain Interference	–0.388^∗∗^	0.374^∗∗^	0.343^∗∗^	0.023	–0.163	–0.066	–0.150	0.029	0.646^∗∗^	–		
(11) HPTo	0.266^∗^	–0.047	–0.317^∗∗^	–0.072	0.021	–0.051	0.067	–0.144	–0.061	–0.205	–	
(12) CPTo	0.222^∗^	–0.021	−0.235^∗^	–0.120	0.012	–0.038	0.206	–0.080	–0.144	–0.035	0.212	–
(13) Pain duration	−0.226^∗^	0.011	0.211	0.036	0.004	0.058	0.123	0.041	0.442^∗∗^	0.264^∗^	–0.028	–0.031

*^∗^*p* < 0.05, ^∗∗^*p* < 0.01. PRS, Pain Resilience Scale; CES-D, Center for Epidemiological Studies Depression Scale; CSQ-R, Coping Strategies Questionnaire-Revised; BPI-SF, Brief Pain Inventory-Short Form; HPTo, Heat Pain Tolerance; CPTo, Cold Pain Tolerance.*

Prior to completing the linear multiple regression analyses below, specific covariates were chosen based upon whether they demonstrated significant associations with key variables of interest including clinical pain severity and interference, HPTo and CPTo, as well as pain coping strategies and pain catastrophizing. Participants’ sex, opioid medication prescription, depressive symptom severity, and chronic pain duration were included as covariates in all study models displayed in Tables 3–5. The reported answer to “Pain Right Now” on the BPI-SF was included as a covariate in all analyses (except for the analysis of pain severity) to control for individual differences in clinical pain severity at the time of study participation.

**TABLE 3:** Multiple regressions models demonstrating associations with pain interference and clinical pain severity.

	**BPI-SF Pain interference**				**BPI-SF Clinical pain severity**			
		
	***R*^2^**	**B**	***SE* B**	**b**	***R*^2^**	**B**	***SE* B**	**b**
**Variables**	0.522^∗∗^				0.251^∗∗^			
Sex^a^		–0.380	0.477	–0.064		–0.199	0.510	–0.040
Opioid prescription^b^		0.651	0.613	0.086		0.214	0.655	0.034
CES-D		0.065	0.020	0.264^∗∗^		0.006	0.022	0.031
Pain Duration		–0.047	0.125	−0.034		0.485	0.122	0.418^∗∗^
Pain Right Now		0.555	0.088	0.555^∗∗^		–	–	–
Pain-Specific Resilience		–0.041	0.018	−0.199^∗^		–0.020	0.019	–0.116

*^*a*^Coded Variable (1 = Males, 2 = Females); ^*b*^Coded Variable (1 = Prescribed, 2 = Not Prescribed). ^∗^*p* < 0.05; ^∗∗^*p* < 0.01. BPI-SF, Brief Pain Inventory-Short Form. CES-D, Center for Epidemiologic Studies Depression Scale.*

### Associations With Clinical Pain Severity and Pain Interference

Table 3 displays the results of two multiple regression models that assessed whether pain-specific resilience was uniquely and significantly associated with clinical pain severity and pain interference reported on the BPI-SF. On the left of Table 3, results revealed that the overall model accounted for a significant 52% of the variance in pain interference [*F*(6, 78) = 14.207, *p* < 0.001]. Greater pain-specific resilience was significantly associated with less pain interference in PLWH and chronic pain even after controlling for covariates (β = −0.199, *p* = 0.022). As seen on the right of Table 3, the overall model did account for a significant portion of variance in clinical pain severity [*F*(5, 79) = 4.320, *p* = 0.002]. However, pain-specific resilience was not significantly associated with clinical pain severity after controlling for covariates (β = −0.116, *p* = 0.283).

### Associations With Pain Coping Strategies and Pain Catastrophizing

A series of three additional multiple linear regressions were conducted to assess whether pain-specific resilience was significantly associated with pain catastrophizing, as well as the use of two pain coping strategies (distraction, coping self-statements). Overall, the multiple regression model presented to the left in Table 4 accounted for a significant 36% of the variance in pain catastrophizing [*F*(6, 78) = 7.143, *p* < 0.001]. Results revealed that greater pain-specific resilience was significantly associated with less catastrophizing about pain (β = −0.309, *p* = 0.002) controlling for covariates. The overall multiple regression model presented in the middle of Table 4 accounted for a significant 13% of the variance in use of distraction [*F*(6, 78) = 2.329, *p* = 0.050]. Greater pain-specific resilience was found to be significantly associated with more frequent use of distraction as a pain coping technique (β = 0.257, *p* = 0.027). As demonstrated on the right side of Table 4, the overall multiple regression model accounted for a significant 18% of the variance in coping self-statements [*F*(6, 78) = 2.891, *p* = 0.013]. Pain-specific resilience was significantly associated with coping self-statements after controlling for covariates (β = 0.233, *p* = 0.039).

**TABLE 4:** Multiple regressions models demonstrating associations with pain catastrophizing, distraction, and coping self-statements.

	**CSQ-R Catastrophizing**				**CSQ-R Distraction**				**CSQ-R self-statements**			
			
	***R*^2^**	**B**	***SE* B**	**b**	***R*^2^**	**B**	***SE* B**	**b**	***R*^2^**	**B**	***SE* B**	**b**
Variables	0.355^∗∗^				0.133^∗^				0.182^∗^			
Sex^a^		–0.052	0.281	–0.017		0.038	0.361	0.011		0.195	0.301	0.068
Opioid prescription^b^		0.001	0.361	0.001		–0.377	0.465	–0.089		–0.359	0.388	–0.098
CES-D		0.047	0.012	0.379^∗∗^		–0.024	0.016	–0.174		–0.023	0.013	–0.191
Pain Duration		0.078	0.074	0.111		0.062	0.095	0.080		0.165	0.079	0.247^∗^
Pain Right Now		0.032	0.052	0.063		–0.077	0.067	–0.012		–0.072	0.056	–0.149
Pain-Specific Resilience		–0.033	0.010	−0.309^∗∗^		0.030	0.013	0.257^∗^		0.023	0.011	0.233^∗^

*^*a*^Coded Variable (1 = Males, 2 = Females); ^*b*^Coded Variable (1 = Prescribed, 2 = Not Prescribed). ^∗^*p* < 0.05; ^∗∗^*p* < 0.01. CSQ-R, Coping Strategies Questionnaire – Revised. CES-D, Center for Epidemiological Studies Depression Scale.*

### Associations With HPTo and CPTo

Results of two multiple regression models examining associations with HPTo and CPTo are presented in Table 5. As shown on the left of Table 5, the overall model accounted for a significant 16% of the variance in HPTo [*F*(6, 78) = 2.426, *p* = 0.033]. Furthermore, results revealed that pain-specific resilience was significantly associated with HPTo, such that participants with greater pain-specific resilience demonstrated higher HPTo (β = 0.302, *p* = 0.009). On the right side of Table 5 it can be seen that the overall model did not account for a significant portion of the variance in CPTo [*F*(6, 78) = 1.149, *p* = 0.342]. Although pain-specific resilience was not significantly associated with CPTo (β = 0.228, *p* = 0.056) after controlling for covariates, there was a trend toward significance. The observed power for the multiple regression model examining associations with CPTo was 0.497.

**TABLE 5:** Multiple regressions models demonstrating associations with heat pain tolerance (HPTo) and cold pain tolerance (CPTo).

	**HPTo**				**CPTo**			
		
	***R*^2^**	**B**	***SE* B**	**b**	***R*^2^**	**B**	***SE* B**	**b**
Variables	0.157^∗^				0.081			
Sex^a^		–1.350	0.491	−0.292^∗∗^		–24.692	26.216	–0.104
Opioid prescription^b^		–0.119	0.632	–0.020		31.685	33.739	0.106
CES-D		0.006	0.021	0.030		0.240	1.127	0.025
Pain duration		0.118	0.129	0.110		4.238	6.891	0.077
Pain Right Now		–0.029	0.091	–0.038		–4.726	4.865	–0.119
Pain-Specific Resilience		0.049	0.018	0.302^∗∗^		1.882	0.969	0.228

*^*a*^Coded Variable (1 = Males, 2 = Females); ^*b*^Coded Variable (1 = Prescribed, 2 = Not Prescribed). ^∗^*p* < 0.05; ^∗∗^*p* < 0.01. CES-D, Center for Epidemiologic Studies Depression Scale.*

### Parallel Multiple Mediation

**FIGURE 1:**
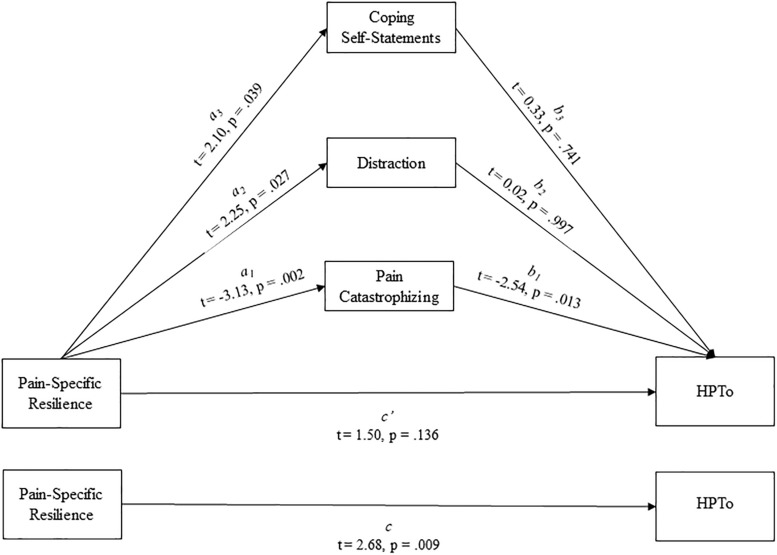
Parallel mediation model depicting the indirect effects of pain-specific resilience on heat pain tolerance (HPTo) through pain catastrophizing, distraction, and coping self-statements.

Whether pain catastrophizing, distraction, and/or coping self-statements significantly mediated the effect of pain-specific resilience on HPTo was examined utilizing a parallel multiple mediation model with bias-corrected bootstrapped confidence intervals (Figure 1). Results indicated that this overall model accounted for a significant 23% of the variance in HPTo (*R*^2^ = 0.229, *p* = 0.015). It was revealed that pain-specific resilience was indirectly related to HPTo (i.e., mediated) through catastrophizing (indirect effect = 0.0168, 95% CI:0.0042 to 0.0354), but not through distraction (indirect effect = 0.0001, 95% CI: −0.0140 to 0.0137) or coping self-statements (indirect effect = 0.0017, 95% CI: −0.0075 to 0.0255). More specifically, the higher HPTo shown by those with greater pain-specific resilience was partly accounted for by their less frequent engagement in pain catastrophizing. Additional parallel multiple mediation models were completed and demonstrated that neither catastrophizing, distraction, nor coping self-statements significantly mediated the effect of pain-specific resilience on CPTo or pain interference.

## Discussion

Resilience to pain is a conceptually complex psychological phenomenon. The previous work of Sturgeon and Zautra (2010) has provided much needed clarity for this topic by addressing important resources and mechanisms that promote pathways to resilience for those with chronic pain. Qualities of an individual and his/her social world such as optimism, perseverance, high socioeconomic status, and a helpful social support network represent resilience resources that increase the likelihood of adaptive responses to chronic pain. Resilience mechanisms refer to the helpful thoughts, affects, and behaviors utilized by individuals with chronic pain when confronting adversity. Resilience resources promote the utilization of beneficial resilience mechanisms, and together these resources and mechanisms interactively influence resilient responses to chronic pain. In this study, the pain-specific resilience measure (Slepian et al., 2016) is arguably an index of resilience mechanisms. This is because its two constituent subscales assess the ability to continue engaging in behaviors or activity when experiencing pain (behavioral perseverance subscale), as well as the ability to maintain positive thoughts and manage negative thoughts or emotions while in pain (cognitive/affective positivity subscale). In this regard, our study demonstrates that PLWH and chronic pain possess wide ranging pain-specific resilience mechanisms that confer either relative protection or vulnerability to the deleterious effects of chronic pain. Those with high pain resilience are perhaps best equipped to cope with chronic pain.

The goal of this study was to investigate the extent to which pain-specific resilience was associated with the following aspects of clinical and experimental pain in a sample of PLWH: (1) engagement in adaptive pain coping strategies, (2) pain catastrophizing, (3) pain interference, and (4) tolerance for painful stimuli delivered in a laboratory setting. As hypothesized, findings suggest that PLWH and chronic pain who demonstrate greater pain-specific resilience may be more likely to engage in adaptive pain coping strategies by specifically utilizing distraction techniques and coping self-statements, while concurrently refraining from catastrophizing about their chronic pain. Additionally, greater pain-specific resilience may mitigate the extent to which chronic pain interferes with daily living and the quality of life of PLWH. Similarly, our findings suggest that greater pain-specific resilience promotes the ability of PLWH and chronic pain to tolerate a painful heat stimulus, an effect which may be attributed to less engagement in pain catastrophizing. Our results are generally consistent with previous studies of chronic pain patients without HIV. For example, greater resilience was associated with better physical functioning and less pain interference in individuals with knee osteoarthritis (Wright et al., 2008). Furthermore, other positive psychological factors associated with resilience such as optimism are indirectly associated with less experimental pain sensitivity via decreased pain catastrophizing (Goodin et al., 2013; Pulvers and Hood, 2013).

Bivariate analyses initially revealed that pain-specific resilience was significantly correlated with diminished clinical pain severity and greater tolerance for a cold pain stimulus; however, these associations were no longer statistically significant after adjustment for covariates in the multiple regression models. It appears that the adjusted multiple regression model examining the unique association between pain-specific resilience and CPTo may have lacked sufficient statistical power to detect a significant association. That the *p*-value was 0.056 and the observed power was 0.497 suggests that with a larger sample size of PLWH and chronic pain, it is very likely the association between greater pain-specific resilience and greater CPTo would have remained significant even after adjustment for covariates. However, future research with a larger sample of PLWH and chronic pain will be necessary to confirm this assertion. In the multiple regression model examining clinical pain severity, the strongest association was with duration of pain. Furthermore, PLWH and chronic pain with the longest pain duration (e.g., >10 years) also reported the lowest levels of pain-specific resilience. These findings suggest that PLWH and chronic pain who have been dealing with their pain for many years may be at greatest risk for poor pain outcomes due to a lack of pain-specific resilience mechanisms.

As a matter of clinical importance, a logical extension of our work would be to address the question of whether a tailored cognitive and behavioral intervention might promote resilience mechanisms, specifically for those PLWH with long duration of pain and who demonstrate low pain resilience. Previous intervention development efforts support the likelihood of this possibility. For example, Padesky and Mooney’s (2012) four-step, strengths-based cognitive-behavioral therapy model was designed to help individuals become more resilient by helping them identify and utilize their personal strengths in ways that promote self-efficacy, positive emotions, and better regulation of negative emotions in response to stress. This strengths-based approach to increasing resilience through cognitive-behavior therapy has not yet been applied to PLWH and chronic pain, to the best of our knowledge. Recent and ongoing work conducted by Merlin et al. (2018) suggests that a tailored and evidence-based behavioral intervention may facilitate adaptation to chronic pain in PLWH by promoting pain-specific resilience mechanisms. In their 12-session pain self-management intervention, “Skills TO Manage Pain (STOMP),” PLWH and chronic pain learn specific skills for coping with stress, building self-efficacy and worth, remaining appropriate engaged in valued activities of daily living. Acquisition of these resilience-building mechanisms is completed in group-based sessions that foster peer support around living with HIV and chronic pain, a resilience resource. Whether an ongoing clinical trial of STOMP will improve the chronic pain experiences of PLWH by promoting pain-specific resilience mechanisms has yet to be determined (clinicaltrials.gov NCT03692611). On balance, future psychological interventions that target pain-specific resilience may play an important role in determining whether PLWH effectively manage and cope with their chronic pain. Cognitive and behavioral-based psychological interventions designed to promote adaptive coping and resilience and specifically tailored for HIV populations may provide patients with the ability to ameliorate distress, reduce pain perception, and increase quality of life (Miller et al., 2019).

This study has several limitations that warrant consideration. First, the cross-sectional design of this study limits our ability to form conclusions regarding whether pain-specific resilience causally yields protective effects against chronic pain in PLWH. Similarly, this study could not address the question of whether pain-specific resilience prevents the worsening of pain interference for PLWH and chronic pain over time. Future longitudinal research will be necessary to better appreciate the mechanistic pathways and processes whereby pain-specific resilience yields its pain protective effects. Second, PLWH in this study completed study questionnaires after completion of the QST battery, not prior. Timing of questionnaire completion could potentially affect how participants respond. Third, our study was not designed with specific focus on possible determinants of pain-specific resilience. Although it appears that some PLWH and chronic pain in this study were especially pain resilient, we cannot meaningfully address how such pain-specific resilience manifested. Future theoretical and applied research seems warranted in both HIV and non-HIV populations to better establish a framework for understanding how pain-specific resilience develops, can be modified, and ultimately protects against the deleterious effects of chronic pain. Whether the previous pain resilience framework previously put forth by Sturgeon and Zautra (2010) applies specifically to PLWH would be a worthwhile investigation. Fourth, the vast majority of our study sample was non-Hispanic Black PLWH who lived below the poverty line. While these sociodemographic factors closely align with the population most affected by HIV in Alabama, and the HIV clinic from which study participants were recruited, the generalizability of our findings may be limited. Future studies should examine whether this study’s findings can be replicated among larger, more diverse populations of PLWH and chronic pain. Findings from our study may prove to be even stronger in subsequent research conducted with PLWH and chronic pain who possess more and better resilience resources such as higher socioeconomic status and deeper social support networks among other. Lastly, we did not specifically assess behavioral domains of pain coping such as exercise and pursuit of hobbies. The CSQ-R measure incorporated in this study asks exclusively about the cognitive domains of pain coping (e.g., distraction, use of self-statements). Therefore, at this time it remains unclear the extent to which pain-specific resilience might be associated with greater utilization of behavioral pain coping strategies in PLWH and chronic pain. Despite these limitations, the results of this study contribute to stronger understanding of how pain-specific resilience might mitigate the deleterious effects of pain for PLWH and chronic pain.

The scant amount of research to date addressing psychological contributors to chronic pain in HIV has largely focused on factors such as pain catastrophizing and depression, which confer vulnerability to negative pain-related outcomes (Scott et al., 2018). More recent studies have begun to also address protective psychological factors that promote resilience and positively influence pain-related outcomes for PLWH (Penn et al., 2019). This study helps to address a gap in the current literature pertaining to the potential impact of positive psychological factors on the experience of chronic pain in PLWH. Individuals with a high degree of pain-specific resilience are generally able to maintain behavioral engagement and appropriately regulate their emotions and cognitions despite prolonged or intense pain (Ankawi et al., 2017). Our findings are consistent with this sentiment given that pain-specific resilience was significantly associated with less pain interference and catastrophizing, more frequent use of adaptive pain coping strategies, and higher tolerance for a painful experimental heat stimulus in a sample of PLWH and chronic pain. Low pain-specific resilience may be an important treatment target in the future for psychologically based chronic pain management. It is encouraging that our findings suggest PLWH and chronic pain may experience improved chronic pain outcomes through the strengthening of pain-specific resilience.

## Data Availability

The datasets generated for this study are available on request to the corresponding author.

## Ethics Statement

This study was carried out in accordance with the recommendations of the National Institutes of Health for the responsible conduct of human subjects research with written informed consent from all subjects. All subjects gave written informed consent in accordance with the Declaration of Helsinki. The protocol was approved by the Institutional Review Board at the University of Alabama at Birmingham.

## Author Contributions

All authors equally contributed to the conceptualization and design of the study. Further, each author was instrumental in helping to analyze the data and to interpret the results, as well as drafting the manuscript and providing the important edits that ultimately culminated with this submission.

## Conflict of Interest Statement

The authors declare that the research was conducted in the absence of any commercial or financial relationships that could be construed as a potential conflict of interest.
